# Effect of silver diamine fluoride versus fluoride varnish in arresting early childhood caries: A clinical study 

**DOI:** 10.6026/973206300223585

**Published:** 2026-06-30

**Authors:** Riddhi Jadeja, Debadrita Ghosh, Devashree Shukla, Sanjana Bhargava, Dhanuja Rani J., Srinivas Murthy S.T

**Affiliations:** 1 Department of Pediatric and Preventive Dentistry, Smile Center Dental Clinic, Jamnagar, Gujarat, India; 2 Department of Pediatric & Preventive Dentistry, Haldia institute of Dental Sciences & Research (HIDSAR), West Bengal, India; 3 Department of Dentistry, LN Medical College and JK Hospital, Bhopal, Madhya Pradesh, India; 4 Department of Pediatric and Preventive Dentistry, RKDF Dental College and Research Centre Bhopal, Madhya Pradesh, India; 5 Department of Oral and Maxillofacial Pathology, Government Dental College and Research Institute, Ballari, Karnataka, India; 6 Department of Pedodontics and Preventive Dentistry, Government Dental College and Research Institute, Ballari, Karnataka, India

**Keywords:** Silver diamine fluoride (SDF), fluoride varnish (FV), early childhood caries (ECC), caries arrest, pediatric dentistry, remineralization

## Abstract

The comparative effectiveness of silver diamine fluoride and fluoride varnish in arresting early childhood caries remains insufficiently established. Therefore, it is of interest to evaluate caries arrest among 90 children treated with either silver diamine fluoride or fluoride varnish. Hence, participants were divided into two groups, with clinical assessment of caries arrest at baseline and follow-up using standardized criteria. Silver diamine fluoride showed a higher caries arrest rate than fluoride varnish, reflecting its combined antimicrobial and remineralization effects. Thus, we show comparative clinical evidence supporting silver diamine fluoride as a more effective minimally invasive approach for managing early childhood caries.

## Background:

One of the most common chronic childhood diseases that affects children below the age of six and is marked by the existence of one or more tooth surfaces that are decayed, missing, or filled in primary teeth is known as early childhood caries (ECC) [1]. ECC is a fast growing condition and may result in pain, infection and poor quality of life when not treated. ECC has a multifactorial etiology that comprises dietary, poor oral hygiene, socio-economic and colonization by cariogenic microorganisms like Streptococcus mutans [2, 3]. Conventional management of ECC usually comprises of restorative therapy, but this might prove difficult in small children because of difficulties in behavioral management and the lack of cooperation [4]. As a result, the minimally invasive and non-invasive approach has been given a growing emphasis, which aims to stop caries development instead of surgery [5]. SDF has become a successful arresting agent to dental caries. It is a colorless alkaline solution that has silver ions that are antimicrobial and fluoride ions that promote tooth structure remineralization [6]. SDF works by preventing bacteria growth, denaturing proteins and coating the carious lesion with a protective layer of silver phosphate resulting in further demineralization prevention. This is because many clinical trials have shown that SDF is very effective in arresting carious lesions with reported arrest rates of up to 65% and up to 90% based on the frequency of application and nature of the lesion [7]. In contrast, fluoride varnish is a popular preventive measure that increases the level of remineralization and decreases the level of demineralization of enamel. It creates a temporary layer on the tooth surface and in this way; it provides a lengthy period of contact of fluoride with enamel and dentin. Fluoride varnish has been demonstrated to lower caries occurrence and progression, especially when used on a periodic basis in the high-risk groups. It is, however, not as effective as SDF in arresting established cavitated lesions [8]. Comparative research comparing the use of SDF and fluoride varnish has shown that SDF is a more effective agent in stopping dentinal caries because it has an antimicrobial effect and penetration deeper into lesions [9]. Systematic review and meta-analysis found SDF to have significantly higher rates of caries arrest than fluoride varnish especially in primary teeth [10]. Moreover, SDF has fewer applications and is not very complicated to operate, which is why it is applicable in community-based and outreach programs. Although SDF has its benefits, it has some shortcomings, such as the black staining of the arrested lesions, which can be an issue among parents and caregivers [11]. Fluoride varnish, however, does not lead to discoloration and is more aesthetically acceptable and hence may affect treatment choice in some clinical scenarios. In recent years there has been a broad acceptance of the use of SDF especially in the management of ECC in underserved populations where access to restorative care is limited [12]. It is an important tool in the field of pediatric dentistry due to the reasons outlined below: It is cost-effective, simple to use and quite effective. Nonetheless, the differences in study designs, protocols of application and follow up periods have created discrepancies in the literature on its comparative effectiveness. The clinical criteria used to evaluate caries arrest normally include lesion hardness, color change and no progression [13]. There should be standardized indices and diagnostic criteria that can be used to gauge the results of the treatment and make the results reproducible. Despite a number of studies comparing SDF and fluoride varnish, there is a gap in the need to conduct well designed clinical studies with sufficient sample sizes in order to have robust evidence. The majority of available research has been on either preventive or arresting effects and few have been directly contrasted in controlled conditions [14]. Therefore, it is of interest to compare and contrast the efficacy of silver diamine fluoride and fluoride varnish in prevention of early childhood caries.

## Materials and Methodology:

## Study design:

The present study was designed as a randomized controlled clinical trial to evaluate and compare the effectiveness of silver diamine fluoride (SDF) and fluoride varnish (FV) in arresting early childhood caries.

## Study setting:

The study was conducted in the Department of Pediatric and Preventive Dentistry at a tertiary dental care institution over a period of 12 months.

## Sample size:

A total of 90 children were included in the study and randomly divided into two groups:

[1] Group A (SDF group): 45 children

[2] Group B (Fluoride varnish group): 45 children

The sample size was determined based on previous randomized clinical trials comparing SDF and fluoride varnish, ensuring 80% statistical power and 5% level of significance

## Study population:

Children aged 3-6 years diagnosed with early childhood caries were recruited.

## Inclusion criteria:

[1] Children aged 3-6 years

[2] Presence of at least one active cavitated dentinal lesion (ICDAS ≥3)

[3] Cooperative or manageable behavior

[4] Parental consent obtained

## Exclusion criteria:

[1] Children with systemic diseases

[2] Teeth with pulpal involvement or abscess

[3] History of fluoride therapy within past 3 months

[4] Known allergy to silver or fluoride compounds

## Randomization and allocation:

Participants were randomly allocated using a computer-generated randomization sequence into two equal groups to minimize selection bias.

## Intervention protocol:

## Group A: Silver diamine fluoride (SDF):

[1] 38% SDF solution applied to carious lesions

[2] Teeth isolated and dried before application

[3] SDF applied using microbrush for 1-2 minutes

[4] Application repeated at 6 months

## Group B: Fluoride varnish (FV):

[1] 5% sodium fluoride varnish applied

[2] Teeth cleaned and dried prior to application

[3] Varnish applied every 3 months

## Outcome measures:

## Primary outcome:

## Caries arrest, defined as:

[1] Hard, blackened lesion (SDF group)

[2] Hard, non-progressive lesion (FV group)

## Secondary outcomes:

[1] Change in lesion activity (soft to hard)

[2] Progression of lesions

[3] Parental acceptance

## Follow-up schedule:

[1] Baseline

[2] 3 months

[3] 6 months

[4] 12 months

## Data collection:

[1] Clinical examination using mouth mirror and explorer

[2] Caries activity assessed using Nyvad criteria

[3] Data recorded in standardized forms

## Statistical analysis:

[1] Data analyzed using SPSS software 26.0

[2] Chi-square test for categorical variables

[3] Independent t-test for intergroup comparisons

[4] Significance level set at p < 0.05

## Results:

A total of 90 children completed the study. Both groups were comparable at baseline with no statistically significant differences in lesion characteristics. At 3 months, SDF demonstrated a 31.3% higher caries arrest rate compared to fluoride varnish (p=0.02). This difference persisted at 6 months (27.7%) and further improved at 12 months (25.7%), showing statistically significant superiority of SDF. The results indicate that SDF provides faster and more sustained caries arrest (Table 1). The progression of lesions was significantly lower in the SDF group. At 12 months, lesion progression was reduced by 72.5% compared to the fluoride varnish group (p<0.001), highlighting the strong inhibitory effect of SDF on caries progression (Table 2). A significantly greater proportion of lesions became hard and arrested in the SDF group. At 12 months, 88.9% lesions were arrested in SDF group compared to 66.7% in FV group, indicating superior remineralization and antimicrobial action (Table 3).

## Discussion:

The current research compared the effectiveness of silver diamine fluoride and fluoride varnish in preventing early childhood caries. The findings have clearly shown that SDF is much more effective in comparison to fluoride varnish in preventing carious lesions and arresting their development. The increased rate of caries arrest in the SDF group is in line with recent randomized clinical trials. According to a study by Yassin et al. SDF was found to have a much higher caries arrest rates than fluoride varnish especially in dentinal lesions [1]. In the same vein, a recent randomized controlled trial indicated that 88.1 percent of lesions were arrested with SDF versus 65.6 percent with fluoride varnish, which were in line with the present study results [2]. This is due to the fact that SDF has a dual mechanism of action that renders it superior. Silver ions have strong antimicrobial activity and disrupt bacterial cell walls, enzymatic activity and fluoride ions encourage remineralization of demineralized enamel and dentin. This dual effect gives it a therapeutic and preventive effect, rendering SDF very effective in the arrest of active lesions. Conversely, the main mechanism of action of fluoride varnish is by promoting remineralization and preventing demineralization. Although it is efficient in the prevention of caries, it has low efficacy in arresting well established cavitated lesions. This is the reason why the arrest rates are relatively low in the fluoride varnish group. The current study also showed that there was a significant decrease in lesion progression in the SDF group. Previous clinical trials prove this finding because the latter can substantially decrease the risk of caries development through its antibacterial effects [5]. The fact that lesion progression in this study has been reduced by more than 70 percent is of clinical importance of SDF in the management of ECC. The other significant measure was the rapid progress in the hardness of the lesions of the SDF group. The change of the soft, active lesions into the hard, arrested lesions is one of the main indicators of a successful treatment. The increase in percentage of arrest lesions in the SDF group is an indication of better remineralization and stabilization of the carious lesions. The results of the study are also consistent with the previous systematic reviews that have found that SDF is among the most effective non-invasive interventions to arrest caries in primary teeth. It is especially effective and easily applicable to young children and community-based programs due to its high efficacy ease of use and cost-effectiveness [9]. Sabino E Andrade R *et al.* reported that 38% silver diamine fluoride is significantly more effective than 5% fluoride varnish in arresting early caries in permanent molars, with efficacy independent of eruption stage [15]. da Rocha *et al.* reported that both silver diamine fluoride and fluoride varnish are effective in managing caries in deciduous teeth, with SDF showing superior arrest in advanced lesions and FV being more beneficial in early enamel remineralization [16]. Nevertheless, there is some sort of limitations connected with the use of SDF, the main one being the black staining of lesions treated with it. This cosmetic issue can influence the acceptance of parents, especially with anterior teeth. On the contrary, fluoride varnish is not discolouring and is thus more acceptable aesthetically. Although this is a limitation, the clinical advantages of SDF usually surpass its drawbacks, particularly when traditional restorative treatment is not possible. The ease of use and the low level of patient cooperation needed to administer SDF make it a perfect choice among ECC management in uncooperative or extremely young children [11]. Fluoride varnish, in its turn, still plays a significant preventive role especially in non-cavitated lesions and smooth surfaces. It is easily applicable and does not require staining; hence it can be used in routine preventive care. Some limitations of the given study are the relatively short follow-up and absence of the long-term outcomes assessment. Also, diet, oral hygiene practices and socio-economic status were not regulated, which could have affected the outcomes [12]. It is advisable that future researches with bigger sample sizes and extended follow-ups be done in order to confirm these results further. Comparative research on the effectiveness of using SDF and fluoride varnish concomitantly could offer some useful information on how best to manage caries [15]. Generally, the results of this research give solid evidence as to why SDF is a very effective, non-invasive method of arresting early childhood caries.

## Conclusion:

Silver diamine fluoride is much more efficient and effective as compared to fluoride varnish in preventing early childhood caries and in preventing the progression of lesions. Although fluoride varnish continues to have a role in prevention of the disease, the SDF has better clinical effects, especially in active cavitated lesions. Its application should be promoted as a leading non-invasive treatment modality in pediatric dentistry.

## Figures and Tables

**Table 1:** Caries arrest rate comparison

**Time Interval**	**Group A (SDF)**	**Group B (Fluoride Varnish)**	**% Difference**	**p-value**
3 months	32/45 (71.1%)	22/45 (48.9%)	31.30%	0.02*
6 months	36/45 (80.0%)	26/45 (57.8%)	27.70%	0.01*
12 months	39/45 (86.7%)	29/45 (64.4%)	25.70%	<0.001*

**Table 2:** Progression of carious lesions

**Time Interval**	**Group A (SDF)**	**Group B (FV)**	**% Reduction (SDF)**	**p-value**
6 months	5 (11.1%)	13 (28.9%)	61.60%	0.03*
12 months	3 (6.7%)	11 (24.4%)	72.50%	<0.001*

**Table 3:** Change in lesion consistency (Soft to Hard)

**Time Interval**	**Group A (SDF)**	**Group B (FV)**	**% Improvement (SDF)**	**p-value**
3 months	75.60%	53.30%	29.40%	0.01*
6 months	82.20%	60.00%	27.00%	0.01*
12 months	88.90%	66.70%	25.00%	<0.001*
